# Unveiling the mechanism of *Pogostemon cablin* (Blanco) Benth in treating heat illnesses via network pharmacology and molecular simulation

**DOI:** 10.1371/journal.pone.0331401

**Published:** 2025-09-08

**Authors:** Xiang Li, Sishi Jiang, Haili Li, Shilin Li, Yingchun Hu

**Affiliations:** Department of Emergency Medicine, The Affiliated Hospital of Southwest Medical University, Luzhou, Sichuan, China; Bowen University, NIGERIA

## Abstract

**Background:**

Heat illness is a dangerous condition marked by a widespread inflammatory response. Although *Pogostemon cablin* (Blanco) Benth and its derivatives are clinically used, their mechanisms remain unclear.

**Methods:**

11 heat illness patients and 14 healthy volunteers from Southwest Medical University Affiliated Hospital were enrolled. Bulk RNA sequencing of peripheral blood samples identified disease-relevant modules via weighted gene co-expression network analysis (WGCNA). Active ingredients and targets of *P. cablin* were retrieved from TCMSP. GO/KEGG, protein-protein interaction (PPI), and ROC analyses were performed. Core genes were localized through single-cell RNA sequencing, with compound-target interactions validated by molecular docking.

**Results:**

Enrichment analysis revealed nine cross-targets in TNF/NF-κB pathways. Core targets (*NFKBIA*, *PARP1*) showed high diagnostic sensitivity/specificity. Single-cell data indicated predominant expression in monocytes and CD1C-CD141 dendritic cells. Molecular docking demonstrated strong affinity of quercetin/quercimeritrin for *NFKBIA*/*PARP1*/*LACTB*, with molecular dynamics confirming structural stability of complexes (RMSD < 2Å after 100 ns).

**Conclusion:**

This pioneering study integrates network pharmacology and molecular simulations to elucidate *P. cablin*’s therapeutic targets for heat illness, providing a foundation for advanced therapies.

## Introduction

Heat illness (HI) represents a clinical spectrum ranging from mild conditions such as heat rash and heat cramps to severe heat stroke [1,2]. Although heat rash and heat cramps are prevalent heat-related disorders, they are not formally classified as severe HI. The primary manifestations of HI include exposure to hot environments or strenuous exercise, accompanied by symptoms of moderate to severe heat stroke [3]. Initial presentations may involve nausea, dizziness, fatigue, headache, profound thirst, and musculoskeletal pain [4]. Without timely intervention, these can progress to heat stroke, characterized by central nervous system dysfunction, multi-organ failure, and circulatory collapse [5]. Studies indicate case fatality rates of 30–80% for patients with high-temperature exposure lacking prompt cooling treatment [6]. Prognosis correlates strongly with hyperthermia severity and duration, underscoring the critical need for early recognition and management [7]. Current clinical protocols prioritize rapid cooling and fluid resuscitation across HI severity levels. Recent research has focused on pathological mechanisms of thermal decompensation, systemic inflammatory responses, and multi-organ failure in severe heat stroke [8]. However, early biomarkers across the HI spectrum and potential targets for traditional Chinese medicine (TCM) interventions remain underexplored.

*P. cablin* (Patchouli) and its derivatives are widely employed in all stages of heat stroke management [9], with patchouli water as a core component. Nevertheless, pharmacological mechanisms underlying patchouli’s protective effects in HI are poorly characterized. Network pharmacology, increasingly applied in TCM research, overcomes limitations of single-target approaches by enabling multi-component analysis. This study systematically investigated patchouli’s active constituents against heat disorders using integrated network pharmacology and molecular docking, bridging traditional knowledge with modern computational methods. Our approach provides novel perspectives for developing natural therapeutics against HI.

## Materials and methods

### Study design flowchart

**Fig 1:**
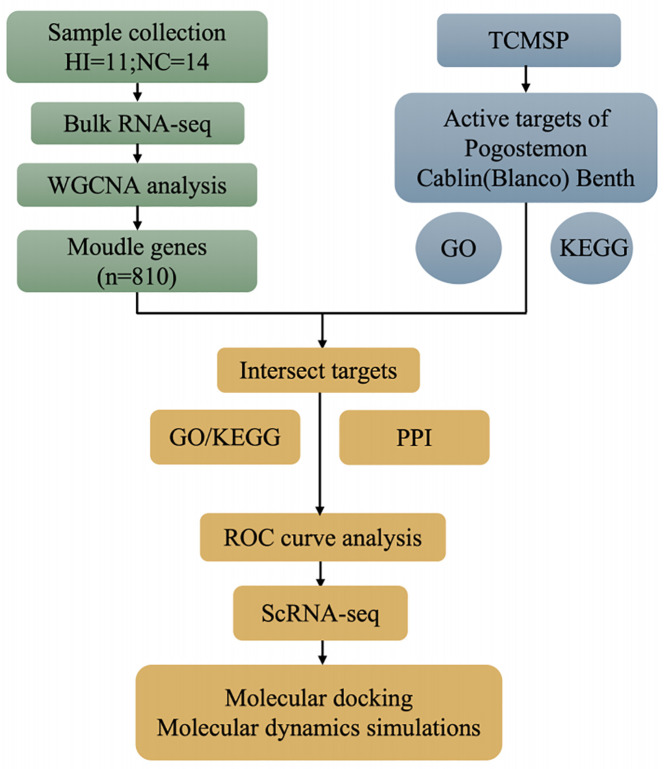
Flowchart for researching.

The overall research workflow is illustrated in Fig 1.

Using a combination of methods including network pharmacology, bulk-RNA-seq, single-cell sequencing, molecular docking and molecular dynamics simulations, we systematically identified how patchouli could target heat illness and its underlying mechanism of action.

### Sample sources and ethics

Approval for the study was given by the Clinical Trials Ethics Committee of our hospital (approval number: ky2023087), in line with medical ethics requirements. Informed consent was signed by all patients or their legal representatives. The China Clinical Trial Registry has registered this trial, and its registration number is ChiCTR 24000084775.

In this study, peripheral blood samples were collected from 11 HI patients and 14 healthy volunteers at the Affiliated Hospital of Southwest Medical University between June and August 2023 using a cohort design. The inclusion criteria were as follows: (1) Adherence to the Diagnostic Criteria for Occupational Heat Stroke (GBZ 41–2019); (2) Patients aged ≥18 years and ≤95 years; (3) Illness duration of less than 24 hours at the time of hospital admission; and (4) Patient or legal representative is willing to participate in the experiment and signs consent form. The exclusion criteria were: (1) patients with a history of organ failure, immune system disorders, or hematological diseases; (2) individuals who declined to participate. During the study, clinical data and inflammatory markers were collected, including age, body temperature, total leukocyte count, neutrophil count, alanine aminotransferase (AST), aspartate aminotransferase (ALT), creatinine, urea, and uric acid. Data were analyzed using SPSS 27.0.1.0, and the independent samples t-test was applied for group comparisons.

### Transcriptome sequences

RNA sequencing serves as a vital method for transcriptome analysis [10]. Total RNA was extracted from peripheral blood samples using TRIzol reagent. Following this, the MGIEasy rRNA kit was employed to remove ribosomal RNA (rRNA) [11]. First-strand cDNA was generated using random primers and reverse transcriptase (MGIEasy kit), and second-strand cDNA was synthesized with DNA polymerase I and RNase H [12]. PCR-amplified products were circularized into single-stranded DNA for DNA nanoball (DNB) generation. After bridge amplification, libraries were quantified using the Agilent 2100 Bioanalyzer [13].

### Data processing

Logarithmic transformation was applied to all sample data using the iDEP 2.01 platform (http://bioinformatics.sdstate.edu/idep/) [14]. Violin plots were generated using GraphPad Prism (version 10.2.3) to assess the homogeneity of the samples. Additionally, Principal Component Analysis (PCA) was conducted to perform preliminary tests on both datasets and identify potential outlier samples. Despite being collected over a two-month period (June–August 2023), all samples were processed uniformly in a single sequencing batch using identical protocols.

### Building the WGCNA network and screening gene modules

WGCNA integrates phenotypic information with gene expression data to identify gene modules associated with the desired traits [15]. The ‘WGCNA’ package in R was utilized to build the co-expression network initially, the gene expression data were clustered and analyzed, with soft thresholds determined by the pickSoftThreshold function. A scale-free topology was approximated by selecting optimal soft thresholds, followed by topological overlap matrix construction. Modules with eigengene dissimilarity <0.25 were merged [16,17], and genes with high expression correlation were assigned to identical modules. Relationships between key modules and traits of interest were further explored by analyzing gene-module correlations (Module Membership, MM) and gene-trait correlations (Gene Significance, GS) to identify potential associations. Finally, module-trait interactions were analyzed for the gene modules with the strongest correlations.

### Active ingredients and objectives

Traditional Chinese Medicine Systems Pharmacology Database (TCMSP) is a comprehensive platform for herbal medicines, providing information on their composition, targets, diseases, and pharmacokinetics [18]. It can be used to analyze the pharmacological effects of herbal medicines and assess their potential value for drug development. In this study, the platform was used to retrieve data on patchouli and screen its active compounds and corresponding targets based on the in vivo ADME (absorption, distribution, metabolism, and excretion) properties, Oral bioavailability (OB) of 30% or higher and drug-likeness (DL) of 0.18 or more were used as the screening criteria. The drug targets were standardized to gene names via the UniProt protein database to ensure data accuracy and consistency.

### Protein-protein interaction networks

Protein-protein interaction networks were constructed using the STRING database (v12.0; https://string-db.org). Intersecting genes between drug targets and heat-related genes were analyzed under the following parameters: species = Homo sapiens, minimum interaction score = 0.4 (medium confidence), and network type = ‘full STRING network’. No additional filters were applied. Resultant networks were imported into Cytoscape (v3.10.1) for topological analysis using CytoNCA and CytoHubba plugins to identify biologically significant hub targets.

### Chinese medicine – Ingredient – Target network

A systematic analysis of the mechanism of Patchouli’s therapeutic effect in treating HI was performed through a comprehensive evaluation of the pharmacological targets of its active ingredients and the identification of key clinical gene modules associated with fever. The overlapping genes were entered into the STRING database to gather the PPI relationships. Finally, the PPI network was visualized and analyzed using Cytoscape software to generate a network diagram that illustrates the core targets and their interactions.

### GO and KEGG pathway enrichment analysis

Three subcategories are part of the Gene Ontology (GO) analysis: Molecular Functions (MF), Biological Processes (BP), and Cellular Components (CC) [19]. The KEGG database, as a comprehensive repository of gene functions, plays a pivotal role in integrating genomic data with higher-order biological processes [20]. To gain insight into the potential biological mechanisms of the target genes of Patchouli, this study employed the OmicShare tool (https://www.omicshare.com/tools/home/report/koenrich.html) [21] to analyze the functional enrichment of Patchouli’s active ingredients and their target genes. This approach enabled mechanistic interpretation of patchouli’s therapeutic effects.

### Receiver operating characteristic curve analysis

A receiver operating characteristic (ROC) curve analysis was performed on the candidate targets with the largest absolute log₂ (fold change) values between the HI and healthy groups. Targets with area under the curve (AUC) values closer to 1 were considered to have high diagnostic value.

### Single-cell RNA sequencing

Following high-throughput sequencing of two peripheral blood samples, the raw reads were analyzed and compared using the official 10x Genomics software CellRanger (version 7.2.0), employing Genome Reference Consortium Human Build 38 (GRCh38) as the reference genome [22]. After the initial quality control in CellRanger was completed, the data underwent further processing and in-depth analysis using the Seurat package (version 3.0.2) in R [23]. The FindAllMarkers function in Seurat identified cluster-specific marker genes. During the analysis, the data were downscaled and visualized using the UMAP algorithm, selected for its capacity to preserve local/global structures in high-dimensional data. Gene expression was visualized using Seurat’s “VlnPlot” and “FeaturePlot”. Additionally, to annotate cell types, expression profiles were correlated with the Human Primary Cell Atlas reference dataset (SingleR package). This involved calculating the correlation between the cell expression profiles and the reference dataset’s cell types, with the highest correlating cell types being assigned to the respective cell populations. Finally, single-cell transcriptomic profiles of heat illness were established.

### Molecular docking

Molecular docking is a structure-based computational simulation method widely used to predict the binding modes of small molecule ligands and their interactions with target protein binding sites [24]. To evaluate binding interactions between patchouli’s key active ingredients and core targets, the compound structures of the nine key active ingredients of Patchouli were retrieved from the PubChem database (https://pubchem.ncbi.nlm.nih.gov), and structures of the target receptor proteins in three dimensions were acquired from the AlphaFold database (https://alphafold.ebi.ac.uk). The compound structures were converted to PDBQT format using OpenBabel (version 3.1.1), and the molecular geometry was optimized using Avogadro. Molecular docking was subsequently performed using the PyRx 0.8 virtual screening tool and AutoDock Vina 1.2.5. During the docking process, the grid box dimensions encompassed the entire protein surface (±5Å from centroid), the exhaustiveness parameter was set to 32, and the conformation with the lowest binding energy was selected for subsequent analysis. Two-dimensional interaction maps of the docking results were generated using Discovery Studio 2019, and the three-dimensional visualization was conducted with PyMOL (version 2.6.0).

### Molecular dynamics simulations

In this study, all-atom molecular dynamics (MD) simulations of protein-ligand complexes, obtained through molecular docking, were performed using GROMACS 2022.4 software. Protein parameterization was carried out using the Amber14SB force field, while the topology files for the small molecule ligands were generated with ACPYPE and Antechamber. The simulated system was constructed within a cubic solvent box, with a minimum distance of 1 nm between the complex and the box edge. To neutralize system charge, sodium and chloride ions were added to the TIP3P water model, and energy minimization was achieved using the steepest descent approach, followed by temperature regulation (300 K) and pressure control (101.325 kPa) using the NVT and NPT ensembles, respectively, to ensure system stability. For each equilibrium phase, 100 ns MD simulations were conducted at 300 K, generating 10,000 frames of trajectory data. Key parameters like root mean square deviation (RMSD), root mean square fluctuation (RMSF), radius of gyration (Rg), and the total hydrogen bonds between the protein and ligand were analyzed from the simulated trajectory files.

## Results

### Subject clinical information

The study included 11 HI patients and 14 healthy controls, who were statistically analyzed for clinical parameters, including age, admission temperature, total leukocyte count, neutrophil count, calcitoninogen, hemoglobin, urea, and alanine aminotransferase. Data for age, body temperature, total leukocyte count, neutrophil count, platelet count, urea, and hemoglobin levels were expressed as the mean ± standard deviation (see Table 1 for details). The findings showed that body temperature and white blood cell count were significantly elevated in patients with heat illness compared to the control group (*p* ≤ 0.05). Additionally, levels of procalcitonin (PCT) and some indices potentially suggestive of organ involvement (e.g., AST, GFR) appeared higher in the HI group, although these differences did not reach conventional statistical significance.

**Table 1:** Subject Clinical Information Form.

Clinical variable	HI (n = 11)	NC(n = 14)	*P* value
AGE(YEAR)	48.73 ± 25.52	36.79 ± 6.28	0.158
TEMPERATURE (°C)	38.39 ± 1.77	36.50 ± 0.51	0.005
WBC (10^9^/L)	10.14 ± 3.81	5.62 ± 1.76	0.003
PCT (NG/ML)	13.97 ± 25.35	0.06 ± 0.07	0.099
LYM (10^9^/L)	1.89 ± 0.76	4.04 ± 8.28	0.403
HGB (G/L)	144.91 ± 23.33	147.79 ± 18.61	0.734
PLT (10^9^/L)	211.09 ± 62.20	240.00 ± 44.90	0.19
NA^+^ (MMOL/L)	138.47 ± 4.18	141.3571 ± 4.57	0.118
K^+^ (MMOL/L)	4.18 ± 0.36	4.51 ± 0.49	0.076
ALT (U/L)	30.32 ± 17.39	31.69 ± 20.19	0.86
AST (U/L)	53.04 ± 43.06	24.91 ± 7.40	0.057
UA (μMOL/L)	448.15 ± 117.71	406.32 ± 101.24	0.349
CR (μMOL/L)	109.39 ± 68.51	75.51 ± 12.50	0.135
GFR (ML/MIN)	86.53 ± 39.09	111.19 ± 12.94	0.068

### Data quality control

**Fig 2:**
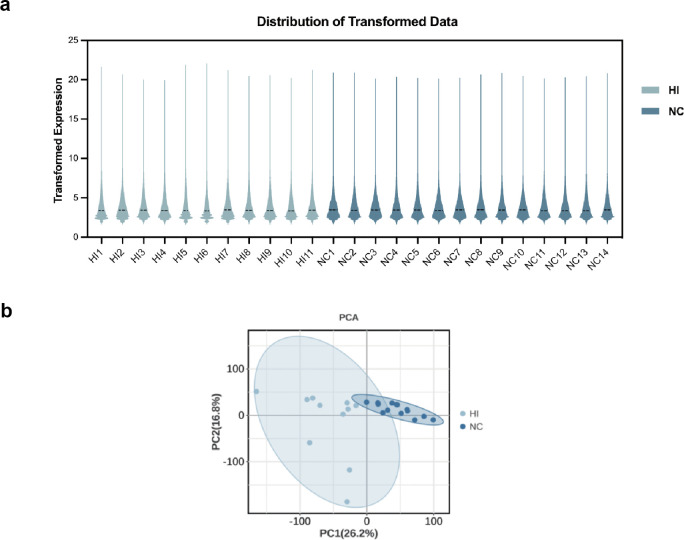
Sample data quality control. (a) A violin plot showing that the data for each sample are distributed at the same level after harmonization. (b) Principal Component Analysis (PCA) of the clustered samples reveals significant differentiation between the group data, with no noticeable outliers.

The logarithmically processed dataset was visualized using violin plots to assess sample homogeneity (Fig 2a). The results showed that the samples from the healthy control and heat illness groups were well-homogenized and exhibited high comparability. Additionally, Principal Component Analysis (PCA) (Fig 2b) revealed clear separation between the two groups in the principal component space, with no outlier samples identified.

### Screening for important genes in HI

**Fig 3:**
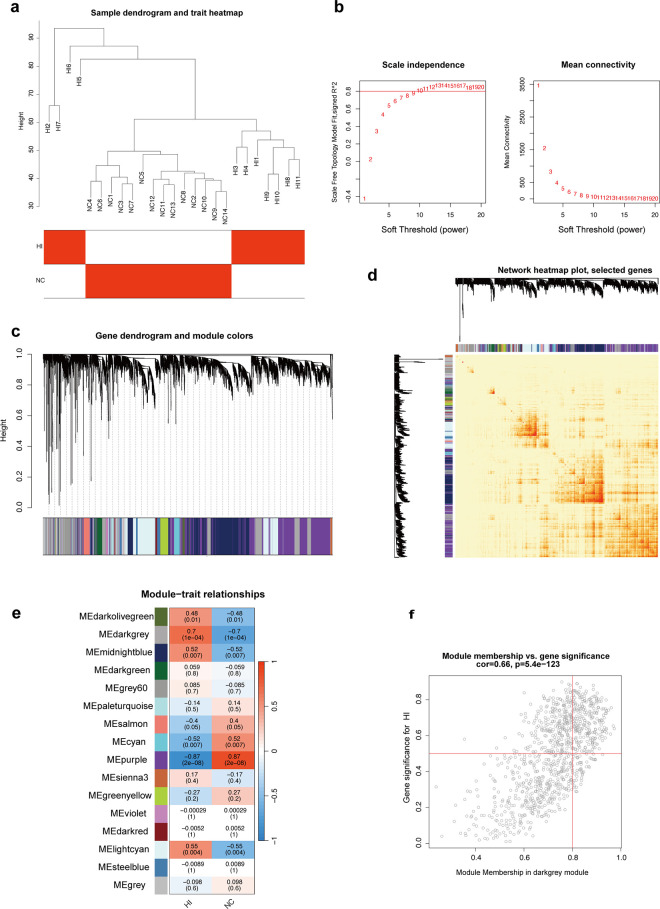
Illustrates the identification of the most relevant modules for HI using weighted co-expression networks. (a) The correspondence between the samples and their grouping information is satisfactory, and no outlier samples were identified after clustering the two groups. (b) The scatter plot corresponding to the power value shows that the trend becomes increasingly linear starting from 12, with average connectivity also becoming more linear when the soft threshold is set to 12. (c) A dendrogram of all genes, organized according to the heterogeneity measure (1-TOM). The color band illustrates the 16 gene modules that were automatically identified and subsequently divided. (d) A heatmap of gene correlations, where darker colors indicate stronger interactions between genes. (e) A correlation plot between module colors and clinical traits is shown below. The darker hue of the heatmap indicates a stronger correlation with the trait, with numerical values within the cells representing the correlation and statistical significance. The black-grey gene module shows the strongest positive correlation with HI. (f) A correlation scatterplot of key modules.

A hierarchical clustering analysis was performed on the sample dataset using the R package ‘WGCNA’ to generate sample dendrograms and phenotypic heatmaps (Fig 3a). The optimal soft threshold was selected using the ‘PickSoftThreshold’ package, with a soft threshold of 12 (R² = 0.83) defined to construct the adjacency matrix (Fig 3b). Genes with highly correlated expression were grouped into the same module after merging modules that were less than 0.25 apart. A gene clustering tree (Fig 3c) and gene module correlation heatmap (Fig 3d) were then constructed. Pearson’s correlation calculated the connections between gene modules and clinical traits, followed by plotting characteristic association maps (Fig 3e). The black and grey module was identified as the most significantly associated with HI (r = 0.7, *P* = 1e-04), containing 810 genes. Phenotypic correlations were further analyzed, generating a scatter plot (Fig 3f) that revealed a strong correlation between GS and MM within the black and grey module (cor = 0.66, *P* = 5.4e-123), indicating that the key genes in this module are closely associated with the trait.

### Analysis of the active ingredients and targets of patchouli

Following the establishment of screening conditions using the TCMSP database, a total of nine principal active ingredients and their corresponding targets were selected for further analysis (Table 2). After removing duplicates, as well as non-human and non-standard targets, 170 effective potential targets were ultimately identified. Subsequently, GO and KEGG enrichment analyses were conducted on these targets.

**Table 2:** Effective active ingredients of patchouli.

MOL ID	Molecule Name	OB (%)	DL
MOL005923	3,23-dihydroxy-12-oleanen-28-oic acid	30.86	0.86
MOL005573	Genkwanin	37.13	0.24
MOL005916	irisolidone	37.78	0.3
MOL005918	phenanthrone	38.7	0.33
MOL005922	Acanthoside	43.35	0.77
MOL002879	Diop	43.59	0.39
MOL000098	quercetin	46.43	0.28
MOL005921	quercetin 7-O-β-D-glucoside	49.57	0.27
MOL005911	5-Hydroxy-7,4’-dimethoxyflavanon	51.54	0.27

**Fig 4:**
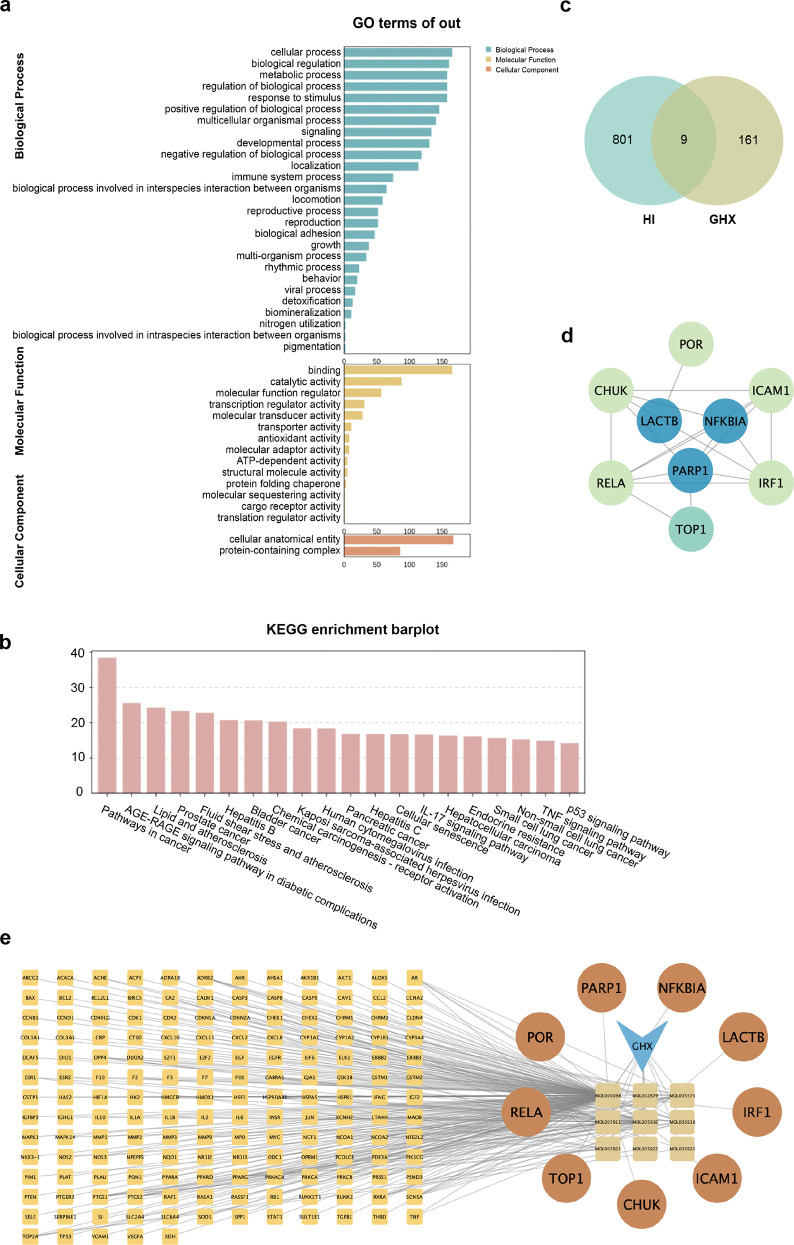
Enrichment analysis of patchouli active ingredients and targets for the treatment of heat illnesses. (a-b) Results of GO and KEGG analysis of the active ingredients of patchouli. (c) Venn diagram showing the intersecting targets between patchouli and HI. The intersection of light green and khaki highlights the common targets. (d) PPI network of the intersecting targets, with core genes identified from the screening highlighted in blue. (e) Traditional Chinese Medicine-ingredient-target network diagram, where the light-yellow matrix on the left represents the 161 targets remaining in patchouli after excluding the intersecting targets. The earthy yellow section on the right corresponds to the 9 cross-targets, while the brown matrix on the far right represents the 9 active ingredients in patchouli. The inverted triangles represent the patchouli compound, and the lines indicate the interactions between each ingredient and its corresponding target.

The results of the GO analysis showed that the BP were primarily enriched in response to oxygenated compound stimuli, cellular response to chemical stimuli, positive regulation of biological processes, and response to lipids. The CC were predominantly localized to extracellular regions, membrane rafts, and membrane microdomains. Additionally, the MF were chiefly related to enzyme binding, binding of transcription factors to DNA, and activity controlled by kinases (Fig 4a). The KEGG pathway enrichment results revealed that these targets are involved in several key biological processes, including the cancer pathway, the AGE-RAGE signaling pathway in diabetes, lipid metabolism, and atherosclerosis (Fig 4b).

Comprehensive analyses suggest that the primary active components of patchouli may exert therapeutic effects through mechanisms such as modulation of oxidative stress, antagonism of external stimuli, and regulation of enzyme activities.

### Protein interaction network

The 170 targets corresponding to the nine active ingredients of patchouli were intersected with the 810 genes identified through WGCNA analysis, resulting in the identification of nine cross-targets (Fig 4c). These cross-targets were considered potential action targets of patchouli in the treatment of HI. Using the STRING database, a PPI network was created and analyzed with Cytoscape software (Fig 4d). Centrality analysis of the nodes was performed using Cytoscape’s plug-in, which served as a reference for screening the core targets. The results suggest that these core targets play critical regulatory roles within the network and may represent key therapeutic targets for HI. Based on these findings, a network diagram was created, illustrating the primary active components of patchouli, their corresponding targets, and their potential interactions (Fig 4e). This visual representation offers a clearer understanding of the relationship between the active ingredients of patchouli and their cross-targets.

### GO and KEGG analyses of intersecting targets

**Fig 5:**
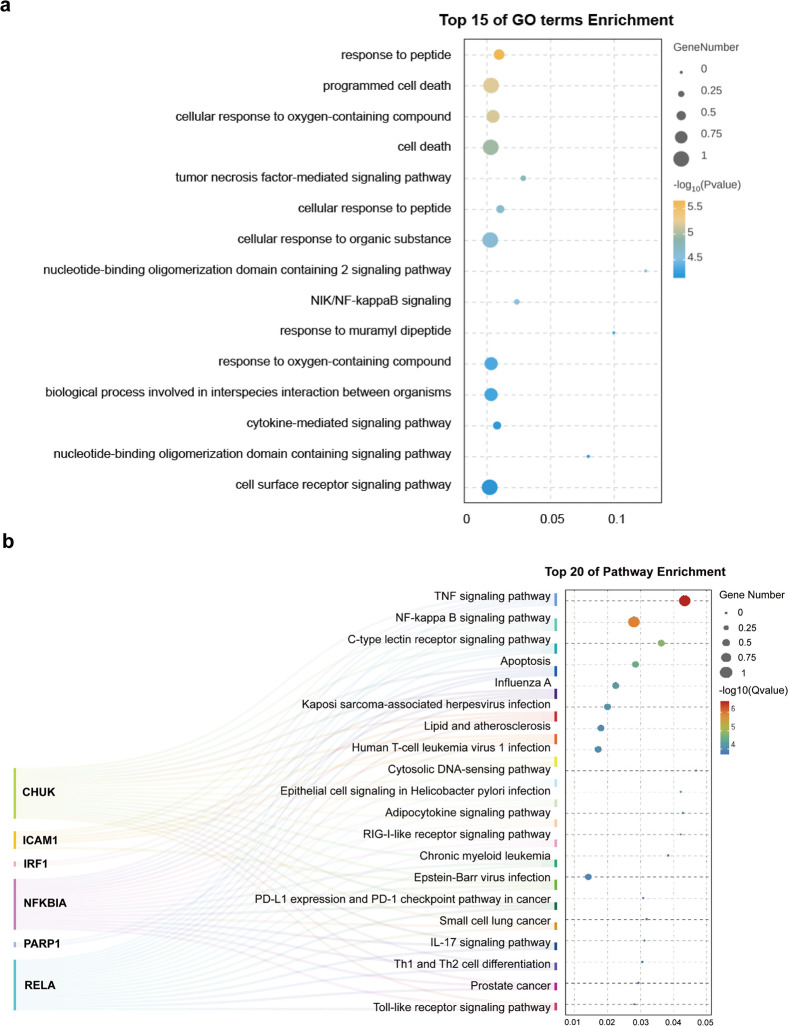
GO and KEGG enrichment analysis of cross-targets. (a) GO enrichment analysis of the cross-targets revealed 15 pathways with significant P values. (b) A pathway enrichment map for the target genes in the top 20 KEGG signaling pathways is shown. The left-side diagram (in mulberry color) illustrates the relationship between the top 20 pathways and their associated target genes. The right-side bubble diagram (arranged from top to bottom) visualizes the pathway enrichment.

The intersecting targets were analyzed through GO annotation, yielding a total of 1,015 gene enrichment data. GO biological process analysis indicated that these target genes were mainly engaged in essential biological processes, including peptide-stimulated response, cell death, cellular response to oxidative compounds, and cellular response to organic substances (Fig 5a). According to KEGG pathway analysis, the relevant genes were significantly enriched in pathways encompassing the TNF signaling pathway, NF-kappa B signaling pathway, C-type lectin receptor signaling pathway, and the pathway of apoptosis.

To further explore the biological implications, the top 20 KEGG-enriched pathways and their corresponding genes were analyzed, resulting in the creation of a pathway enrichment bubble map (Fig 5b). Six primary targets were identified in the analysis, which are closely related to the regulation of inflammation and apoptosis, implying that these targets could be essential in the anti-inflammatory action of patchouli when treating heat illness.

### Potential genes associated with HI diagnosis

**Fig 6:**
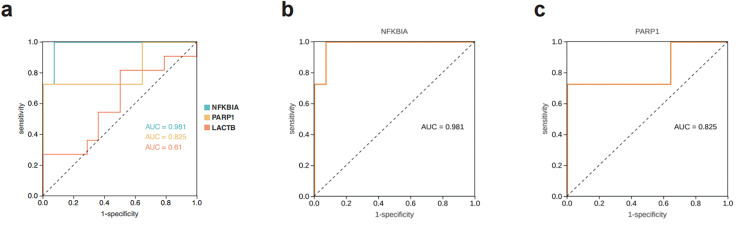
ROC curve diagram. (a) The sample dataset was used to generate an ROC curve, where the AUC value of each target reflects the correlation between the gene and the manifestation of HI. (b-c) show the AUC values for *NFKBIA* and *PARP1*, respectively.

In this study, potential genes located at the center of the PPI network were selected for further characterization. The AUC values for each gene are as follows (Fig 6a): *NFKBIA* (0.981), *PARP1* (0.825), and *LACTB* (0.61). Higher AUC scores indicate a greater ability to distinguish between patients with and without the disease. The results suggest that *NFKBIA* and *PARP1* (Fig 6b-6c) may have high sensitivity and specificity for heat illness, with *NFKBIA* exhibiting the highest diagnostic sensitivity.

### Single-cell RNA sequencing analysis

**Fig 7:**
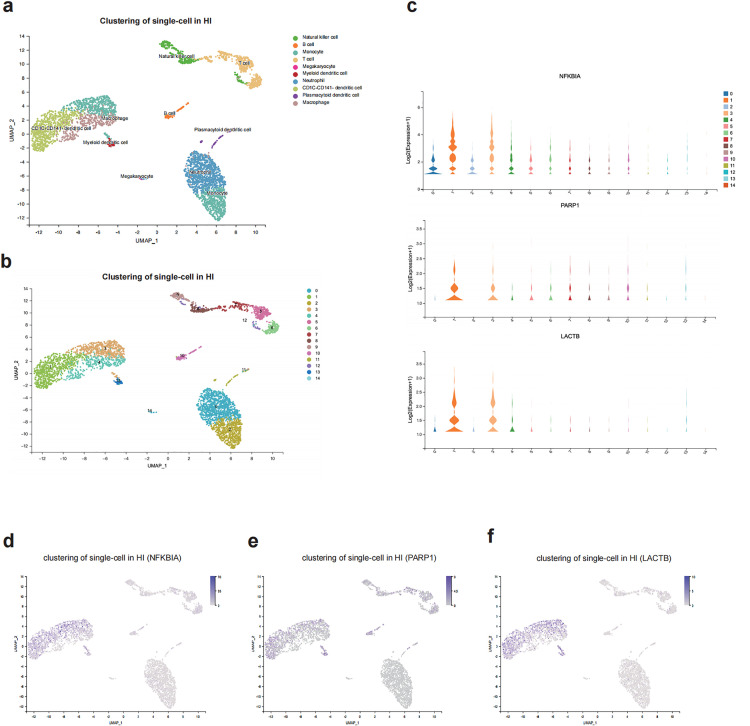
Single-cell sequencing localization. (a) Cell type distribution of the heat illness group samples visualized by UMAP plots, highlighting the clustering and distribution characteristics of different cell groups in two-dimensional space. (b) UMAP clustering into 14 clusters. (c) Violin plots of gene expression. The violin plots illustrate the expression patterns of specific genes across different cell clusters. The vertical axis represents the proportion of cells expressing the gene in a given cell lineage, while the horizontal axis corresponds to the classification of cell clusters. (d-f) Distribution of expression levels of *NFKBIA, PARP1, and LACTB* in peripheral blood cells. Gene expression levels are represented by color gradients, with lighter colors indicating lower expression and darker colors indicating higher expression, visually reflecting the abundance of these genes across different cell clusters.

After single-cell sequencing of the two sample sets, cells were classified into 10 relevant subpopulations through downscaling and clustering analysis (Fig 7a). Based on the expression profiles of marker genes, major cell types were identified, comprising B cells, T cells, monocytes, macrophages, neutrophils, natural killer cells, and CD1C-CD141 dendritic cells (Fig 7b). Further analysis revealed that the expression levels and proportions of genes of interest varied across different cell clusters (Fig 7c), providing a critical foundation for the subsequent exploration of related mechanisms.

The marker genes *NFKBIA*, *PARP1*, and *LACTB* exhibited high expression in monocytes and CD1C-CD141 dendritic cell lineages, primarily localized to cell clusters 1 and 3 (Fig 7d-7f). Additionally, *NFKBIA* was notably expressed in macrophage and neutrophil clusters, while *PARP1* showed high expression in clusters 7 and 10, corresponding to T-cell and B-cell populations, respectively. *LACTB* was also prominently expressed in macrophage populations. These results imply that the marker genes could be crucial in various immune cell groups, possibly influencing the molecular mechanisms underlying the effects of patchouli in the treatment of HI.

### Molecular docking validation of active ingredients

**Fig 8:**
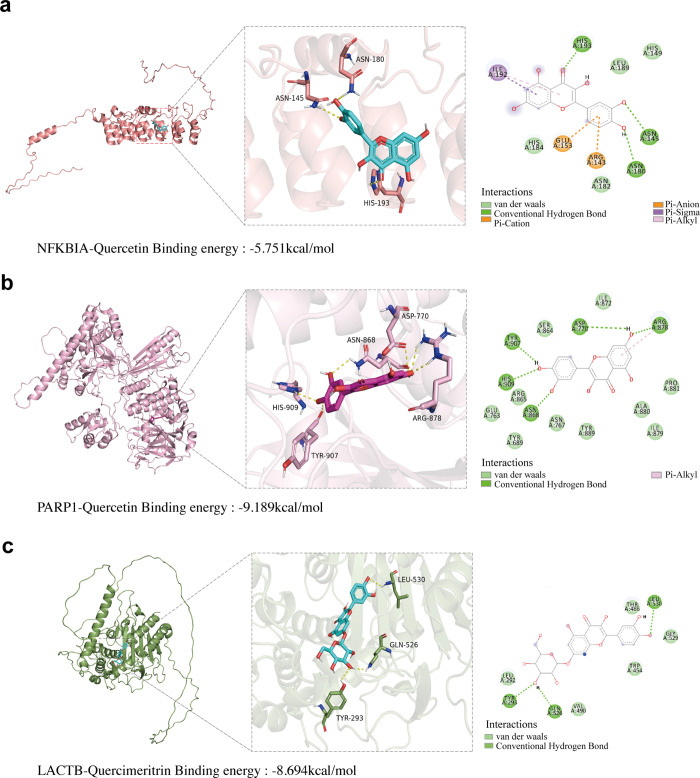
Receptor and ligand binding visualization. (a-c) Images depicting the 3D structures of *NFKBIA*, *PARP1*, and *LACTB*, along with detailed views of the docking interactions between the small molecule compounds and these proteins, highlighting the specific interactions formed. The analysis revealed that *NFKBIA* (HIS193, ASN180, ASN145) interacts with quercetin via three hydrogen bonds, with bond lengths of 2.48 Å, 2.82 Å, and 2.66 Å, respectively. *PARP1* (HIS909, TYR907, ASP770, and ARG878) forms five hydrogen bonds with quercetin. Additionally, *LACTB* (TYR293, GLN526, LEU530) forms three hydrogen bonds with quercimeritrin, with bond lengths of 2.46 Å, 1.98 Å, and 2.66 Å, respectively.

PyRx is a virtual screening tool for drug discovery that focuses on exploring the binding properties of small molecules interacting with biological targets [25]. Molecular docking analysis was performed in this study using the AutoDock Vina module in PyRx to analyze the binding sites of the key active ingredients (quercetin and quercimeritrin) with target proteins and their binding energies (Fig 8a-8c).

The results of virtual screening indicated that the binding energies of patchouli’s active components to potential targets ranged from −5.751 to −9.189 kcal/mol, suggesting strong affinity for the target proteins. Specifically:

- Quercetin formed five hydrogen bonds with *PARP1* (residues HIS909, TYR907, ASP770, ARG878) at −9.189 kcal/mol
- Quercimeritrin formed three hydrogen bonds with *LACTB* (residues TYR293, GLN526, LEU530) at −8.694 kcal/mol
- Quercetin formed three hydrogen bonds with *NFKBIA* (residues HIS193, ASN180, ASN145) at −5.751 kcal/mol

These findings highlight the strong binding affinity of patchouli’s active ingredients for specific targets, providing molecular evidence to support their potential pharmacological effects.

### Molecular dynamics simulation

During the process of ligand-receptor binding, a series of conformational changes typically occur. In the event of a minor structural rearrangement, the ligand may gain access to the binding site via a relatively minor displacement. Conversely, if the magnitude of the conformational change is considerable, the protein structure may undergo a significant alteration [26]. To address such flexibility issues, molecular dynamics simulation (MD) represents an effective means of doing so [27]. Prior to molecular docking, MD simulations can assist in the extraction of a range of novel and extensive protein conformations from simulation trajectories. These conformations can then be employed to generate more accurate structural models for subsequent docking. Furthermore, MD simulations can be utilized following docking to optimize the structure of the complexes, to compute interaction energies in greater detail, and to elucidate the mechanism of ligand binding in greater depth. In order to evaluate the stability of ligand-receptor binding, MD simulations were performed on the *NFKBIA*-Quercetin, *PARP1*-Quercetin, and *LACTB*-Quercimeritrin complexes.

**Fig 9:**
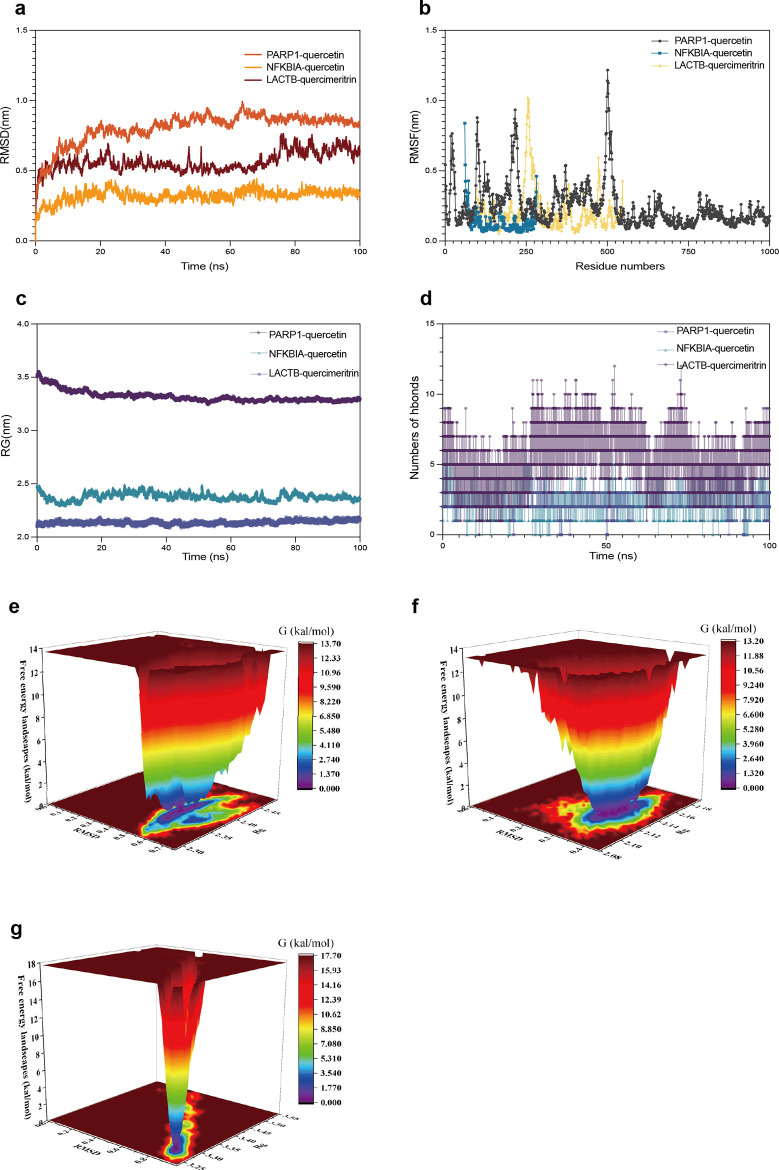
Molecular dynamics simulation of protein-monomer. (a) RMSD plots of the simulated protein-ligand binding for the three complexes, with smaller RMSD values indicating less structural deviation. The RMSD values for the aforementioned complexes remained within 0.2 nm, suggesting that the proteins maintained stable conformations without significant deformation. (b) RMSF plots for the *NFKBIA*-quercetin, *PARP1*-quercetin, and *LACTB*-quercimeritrin complexes. (c) The Rg for the three protein-ligand complexes exhibited minimal fluctuations within the 0-100 ns interval, indicating that the complexes retained structural stability. (d) A plot showing the number of hydrogen bonds formed between the three protein-ligand complexes over time, with a higher number of hydrogen bonds indicating a stronger binding affinity. (e) Gibbs free energy landscape for the *LACTB*-quercimeritrin complex. (f) Gibbs free energy landscape for the *NFKBIA*-quercetin complex. (g) Gibbs free energy landscape for the *PARP1*-quercetin complex.

Root Mean Square Deviation (RMSD) is a mathematical metric that is commonly used to assess differences between structures, simulation results, or datasets. It measures the spatial deviation of each atom in a set of structures. Fig 9a shows the RMSD of the *NFKBIA*-Quercetin, *PARP1*-Quercetin, and *LACTB*-Quercimeritrin complexes as a function of RMSD over time. In this analysis, the complete structure of the complex was used. The results demonstrated that the RMSD curves of the *NFKBIA*-Quercetin complex exhibited a relatively stable profile between 20 and 100 ns, whereas the RMSD curves of the *PARP1*-Quercetin complex reached a stable state after 40 ns. The *LACTB*-Quercimeritrin complex demonstrated sustained stability between 0 and 100 ns. In this simulation, the RMSD of the ligand exhibited fluctuations within the range of approximately 0.2 nm, with minimal structural alterations within the protein binding pocket. Additionally, there were no discernible instances of significant detachment of the ligand from the binding site.

Root Mean Square Fluctuation (RMSF) is employed to quantify the range of fluctuation exhibited by each residue within a protein, thereby reflecting the flexibility of the local structure. Regions with higher RMSF typically represent more flexible parts of the protein, such as active sites and ligand binding pockets. In contrast, regions with lower RMSF are usually more stable, as illustrated in Fig 9b, which depicts the fluctuation of each residue in the complex. The *NFKBIA* complex exhibits high fluctuation in the residue 61–283 region, indicating that this region is more flexible. Similarly, the *LACTB* complex displays high fluctuation at residues 240–270, suggesting that this region is also highly flexible. Additionally, the *PARP1* complex demonstrated high flexibility at multiple residues.

The gyration radius is a global feature of protein structure, defined as the average distance between the centre of mass of a protein and its atoms. The radius of gyration serves as an important indicator of the overall folding and compactness of the protein. A small radius of gyration typically indicates that the protein is compact and tightly folded, whereas a large radius of gyration may suggest that the protein is in an unfolded state or has undergone changes in structural looseness or instability during the simulation process. Fig 9c illustrates the change in radius of gyration over time in three sets of molecular dynamics simulations. The radius of gyration remained essentially stable throughout the course of the simulations for all three complexes, indicating that the protein conformation was stable and did not undergo significant changes.

Hydrogen bonding represents a common non-covalent interaction observed in protein-ligand complexes, and it typically exerts a significant influence on the stability and affinity of molecular binding. The number of hydrogen bonds in protein-quercetin or quercetin complexes can be used as an indicator of their binding strength. Fig 9d illustrates the trend in the number of hydrogen bonds over time. The *PARP1*-quercetin complex exhibited the highest hydrogen bond density and binding strength, which was consistent with the binding energy results obtained in the previous molecular docking analyses. This further verified the stronger binding ability of quercetin and *PARP1* complexes.

The Free Energy Landscape (FEL) plot depicts the distribution of free energy derived from RMSD and Rg calculations performed during molecular dynamics simulations of proteins and ligands (Fig 9e-9g). A color gradient is employed in the illustration to indicate the degree of free energy, with blue and purple regions denoting low free energy regions, signifying that the system is more stable at these positions and is most likely to exist in these regions. The graphical results demonstrate that all three groups of complexes are capable of maintaining a stable state in the vicinity of the binding site throughout the molecular simulation, thereby corroborating their binding stability to the receptor during the simulation.

In this study, the binding characteristics and structural stability of three protein–compound complexes (*NFKBIA*–quercetin, *PARP1*–quercetin, *LACTB*–Quercimeritrin) were systematically evaluated through 100 ns MD simulations. All complexes exhibited stable binding conformations with no significant deviations in backbone RMSD, and their interactions were primarily maintained through non-covalent forces such as hydrogen bonding and hydrophobic contacts.

RMSF analysis further revealed several residues playing stabilizing roles within the dynamic protein–ligand interfaces. In the *NFKBIA*–quercetin complex, residues ASN180 and HIS193 showed moderate flexibility (RMSF > 0.85 Å), suggesting localized mobility that allowed better adaptation to ligand binding while preserving global structural integrity. For *PARP1*, a flexible segment spanning residues 498–505 was identified, with key residues HIS909 and TYR907 located adjacent to this region; these may contribute to dampening local fluctuations through stabilizing contacts with the ligand. In the *LACTB*–Quercimeritrin complex, residues TYR293 and LEU530, located within a loop spanning residues 251–260, exhibited higher RMSF values (>1.0 Å) yet maintained consistent interactions with the ligand, implying dynamic but structurally supported binding under simulated heat stress conditions.

These findings suggest that specific amino acid residues in all three complexes contribute to maintaining local flexibility while stabilizing the ligand-binding interface—supporting the robustness and biological plausibility of the predicted interactions.

## Discussion

The heat illness continuum represents a range of case states, encompassing a spectrum of conditions from heat exhaustion and heat injury to heat stroke. This continuum covers a range of conditions, from mild heat stroke to highly lethal pyrexia. The clinical manifestations of these diseases in their early stages are somewhat similar, which presents a challenge for differential diagnosis of different degrees of HI. Concurrently, the precise pathological mechanisms underlying HI remain incompletely elucidated. Current research suggests that processes like inflammation, oxidative stress, and cell death are crucial in the development and advancement of febrile illnesses [28]. Furthermore, other factors, including iron death, myocardial metabolic disorders and heat stroke protein (HSP) dysfunction, are also believed to be closely associated with the multi-organ functional damage caused by HI [29]. The transition from heat stress to HI is significantly characterized by excessive acute-phase and inflammatory responses, and early intervention is essential to improve disease progression and prognosis. Therefore, the inflammatory response and oxidative stress, and their related cell signaling pathways, are considered to be important regulatory aspects in the treatment of severe HI. The in-depth study of these mechanisms not only helps to reveal the pathophysiological basis of HI, but also provides a potential direction for exploring new therapeutic strategies.

In this study, we systematically identified HI-associated disease genes using peripheral blood samples from HI patients, combined with RNA sequencing and weighted gene co-expression network analysis. *NFKBIA* and *PARP1* were identified as potential biomarkers with high diagnostic sensitivity through bioinformatics analysis. These findings not only provide an important foundation for the study of the molecular mechanism of HI, but also provide a potential reference for early intervention of the disease and improvement of patient prognosis. Due to the study’s small sample size and the absence of independent validation from a public dataset, the actual diagnostic value of the markers has not been completely determined. Nonetheless, the results of this study lay a theoretical foundation for subsequent larger multicenter studies exploring new directions for HI early diagnosis and treatment.

The practice of TCM employs a holistic and systemic approach to the prevention and treatment of diseases, with its multi-component, multi-target, and multi-pathway characteristics offering significant potential for managing heat exposure conditions. It has been demonstrated that patchouli possesses anti-inflammatory, antibacterial, and immunomodulatory effects, and its various active components exhibit remarkable pharmacological properties [30,31]. For instance, patchouli alcohol has been shown to effectively reduce inflammatory diseases by suppressing pro-inflammatory factor expression and boosting reducing agent levels [32]. Furthermore, research by Wang et al. indicates that β-PAE is vital for anti-inflammatory properties by maintaining cytokine balance [33].

The presented research examples exemplify the significant role of patchouli’s active components in inflammation regulation, providing a robust scientific foundation for potential applications in inflammatory diseases. A search of public Chinese medicine and gene databases was conducted to identify patchouli’s main active components and their targets. Gene enrichment analysis indicated that patchouli may act by modulating oxidative stress and enhancing resistance to external stimuli, corroborating previous research on febrile diseases. Nevertheless, systematic studies on patchouli for febrile illnesses remain scarce. Our study offers novel insights into patchouli-based natural remedy development.

Quercetin is a naturally occurring flavonoid exhibiting notable antioxidant and anti-inflammatory properties. The 2,3-double bond in its structure forms a specific reducing structure with the 4-oxygen bond on the C-ring, endowing it with strong antioxidant capacity [34–39]. Quercetin exerts anti-inflammatory effects through multiple cell signaling pathways. A study by Aboud et al. demonstrated quercetin-laser therapy reduces pro-inflammatory cytokine expression [40]. Rao et al. demonstrated quercetin inhibits LPS-induced tumor necrosis factor-α (TNF-α) and nitric oxide production in macrophages. Moreover, it reduces neutrophil migration by inhibiting actin polymerization. Zheng and Wu observed quercetin diminishes cytokine release and alleviates inflammatory responses by inhibiting NF-κB and IL-17 pathways [41]. Specifically, quercetin targets NF-κB and Akt pathways, hindering binding to IP-10 and MIP-2 promoters [42].

Quercimeritrin has been demonstrated to exhibit good water solubility and significant anti-inflammatory and antioxidant potential [43]. Its structural configuration endows free radical scavenging capacity, ability to reduce oxidative stress and inhibit pro-inflammatory factors. Recent studies demonstrate quercetin derivatives in Moringa leaves exhibit antibacterial activity against Pseudomonas aeruginosa [44]. Moreover, it may exert anti-inflammatory effects by inhibiting NF-κB signaling. These studies support Quercimeritrin utilization for inflammatory and infectious conditions involving oxidative stress.

However, there is paucity of evidence suggesting quercetin derivatives exert therapeutic effects on HI through these targets. This study employed molecular docking and dynamics simulations to confirm quercetin and its derivative bind *NFKBIA*, *PARP1*, and *LACTB*, showing stable binding interactions. These findings indicate these targets may serve as crucial molecular foundations for HI treatment.

Poly (ADP-ribose) polymerase 1 (*PARP1*) is a crucial enzyme essential for DNA repair [45]. Sydney Shall and colleagues first demonstrated *PARP1* mediates DNA damage repair [46], and that it exerts its effects through a synergistic mechanism involving its three structural domains, which enable it to re-spond to a range of DNA damage types [47–49]. Inhibition of *PARP1* has emerged as a highly effective strategy for the clinical treatment of cancer [50]. Previous studies demonstrate *PARP1* inhibition substantially mitigates necrosis in renal ischemia-reperfusion injury models [51]. The genetic material of patients with HI is frequently subjected to a multitude of toxic stresses that cause DNA damage, underscoring the vital importance of DNA repair.

In this study, we employed molecular docking analysis to investigate the potential of quercetin, an active ingredient of patchouli, to regulate DNA damage repair by targeting *PARP1* and inhibiting its activation, thereby influencing cellular outcomes following acute oxidative or inflammatory injury. Notably, single-cell transcriptomic analysis revealed high expression of *PARP1* in monocytes and dendritic cells, suggesting that, beyond its role in DNA repair, *PARP1* may also modulate immune activation and cytokine signaling during heat illness through these innate immune populations.

IκBα, or nuclear factor κB inhibitor α (*NFKBIA*), is responsible for negatively regulating NF-κB signaling, thereby inhibiting its activation [52]. *NFKBIA* inhibits pro-inflammatory cytokine expression through competitive binding to the RHD domain of NF-κB [53]. While quercetin does not impede TNF-induced RELA phosphorylation and IκBα degradation, it inhibits IκBα phosphorylation by targeting IKKα phosphorylation, preventing NF-κB activation [54,55]. This study found quercetin binds *NFKBIA*, blocking NF-κB signaling through *NFKBIA* activation. Single-cell data showed *NFKBIA* highly expressed in monocytes and neutrophils, suggesting it may act as a key inflammation suppressor in innate immune cells during HI development, potentially regulating inflammatory responses.

*LACTB* is an active serine protease in the mitochondrial intermembrane space, vital for lipid metabolism regulation [56]. Co-expression analysis indicated *LACTB* influences cellular functions by modulating metabolic circuits [57]. Recent studies demonstrate LACTB inhibits liver cancer progression by regulating ferroptosis [58]. Ferroptosis, characterized by iron-dependent lipid peroxide buildup, links to numerous diseases [59]. In HI studies, ACSL4 (a key ferroptosis enzyme) was identified as crucial in exertional heat stroke progression to rhabdomyolysis [60]. Additionally, TLR4 mitigates heat stroke-induced myocardial injury by suppressing inflammation and ferroptosis [61]. cPLA2-mediated ferroptosis inhibition may alleviate heat stroke-induced acute liver injury [62]. These findings suggest targeting *LACTB* with quercimeritrin may be a promising research area. Single-cell RNA sequencing revealed *LACTB* predominantly expressed in macrophages, suggesting potential involvement in regulating immune cell metabolism under heat stress.

This work employs modern bioinformatics techniques to explore patchouli constituents (Quercetin/Quercimeritrin) for HI treatment. This study employs molecular docking and WGCNA to identify HI-associated targets. The potential of herbal components in cell signaling and immune regulation was demonstrated through network pharmacology and molecular docking techniques.

Despite the promising findings, this study has certain limitations. Due to the absence of experimental validation for key targets such as *NFKBIA* and *PARP1*, the conclusions should be regarded as exploratory. Future studies will aim to clarify the specific roles of these targets in heat illness–related signaling pathways through detailed molecular and cellular experiments.

## Conclusion

An integrated approach combining bioinformatics, network pharmacology, molecular docking, and molecular dynamics simulation was employed to comprehensively analyze the potential role of patchouli in the treatment of HI. The findings revealed that the primary active constituents of patchouli (quercetin and quercetin glycosides) exhibit high affinity for pivotal proteins involved in inflammatory and immune responses (*NFKBIA*, *PARP1*) and the ferroptosis-related protein *LACTB*. Molecular dynamics simulations confirmed the stability of the protein-ligand complexes. Gene enrichment analysis of these targets revealed pathways associated with TNF signaling, NF-kappa B signaling and ferroptosis, which are pivotal for HI pathophysiology, supporting the proposed therapeutic effects of patchouli. Furthermore, these findings establish a mechanistic foundation for future studies on patchouli’s anti-HI mechanisms.

## Supporting information

S1 Table Initial data of the study. (XLSX)
